# Veterans’ ambulatory care experience during COVID-19: veterans’ access to and satisfaction with primary care early in the pandemic

**DOI:** 10.1186/s12875-022-01851-3

**Published:** 2022-09-21

**Authors:** Brice Thomas, Aanchal Thadani, Patricia V. Chen, Israel C. Christie, Lisa M. Kern, Mangala Rajan, Himabindu Kadiyala, Drew A. Helmer

**Affiliations:** 1 Baylor College of Medicine, Houston, USA; 2 University of North Carolina Hospitals, 101 Manning Drive, CB# 7160, Chapel Hill, USA; 3 Michael E. DeBakey VA Medical Center, Houston, USA; 4 Weill Cornell Medicine, New York, USA

**Keywords:** Access, COVID-19, Primary care, Pandemic, Satisfaction, Veterans

## Abstract

**Background:**

The COVID-19 pandemic caused widespread changes to healthcare, but few studies focus on ambulatory care during the early phase of the pandemic. We characterize veterans’ ambulatory care experience, specifically access and satisfaction, early in the pandemic.

**Methods:**

We employed a semi-structured telephone interview to capture quantitative and qualitative data from patients scheduled with a primary care provider between March 1 – June 30, 2020. Forty veterans were randomly identified at a single large urban Veterans Health Administration (VHA) medical center. The interview guide utilized 56 closed and open-ended questions to characterize veterans’ perceptions of access to and satisfaction with their primary care experience at VHA and non-VHA primary care sources. We also explored the context of veterans' daily lives during the pandemic. We analyzed quantitative data using descriptive statistics and verbatim quotes using a matrix analysis.

**Results:**

Veterans reported completing more appointments (mean 2.6 (SD 2.2)) than scheduled (mean 2.3 (SD 2.2)) mostly due to same-day or urgent visits, with a shift to telephone (mean 2.1 (SD 2.2)) and video (mean 1.5 (SD 0.6)). Among those who reported decreased access to care early in the pandemic (*n* = 27 (67%)), 15 (56%) cited administrative barriers (“The phone would hang up on me”) and 9 (33%) reported a lack of provider availability (“They are not reaching out like they used to”). While most veterans (*n* = 31 (78%)) were highly satisfied with their VHA care (mean score 8.6 (SD 2.0 on a 0–10 scale), 9 (23%) reported a decrease in satisfaction since the pandemic. The six (15%) veterans who utilized non-VHA providers during the period of interest reported, on average, higher satisfaction ratings (mean 9.5 (SD 1.2)).

Many veterans reported psychosocial effects such as the worsening of mental health (*n* = 6 (15%)), anxiety concerning the virus (*n* = 12 (30%)), and social isolation (*n* = 8 (20%), “I stay inside and away from people”).

**Conclusions:**

While the number of encounters reported suggest adequate access and satisfaction, the comments regarding barriers to care suggest that enhanced approaches may be warranted to improve and sustain veteran perceptions of adequate access to and satisfaction with primary care during times of crisis.

**Supplementary Information:**

The online version contains supplementary material available at 10.1186/s12875-022-01851-3.

## Background

The COVID-19 pandemic massively impacted life in the United States, leading to unprecedented disruptions to daily life and over 900,000 deaths as of April 2022 [1]. Early in the pandemic in the United States (March – June 2020), in the face of growing infections, hospitalizations, and deaths, there was tremendous uncertainty about the transmissibility and virulence of the SARS-CoV-2 virus and the effectiveness of risk mitigation measures (e.g., face masks), no vaccine, and no proven treatments. This prompted closures, curtailment of services, and a shift from in person to virtual interactions in most domains of daily life.

The pandemic also impacted health care utilization in all settings. For example, 7.3 million VHA appointments were cancelled from March 15 through May 1, 2020, thirty-two percent of which had no indication of follow up or tracking [2]. The use of telehealth rapidly increased to address needed care [3], more than one in three adults (36%) reported delaying or foregoing care due to worry about exposure or lack of services [4]. In addition to deaths directly caused by COVID-19, delays in care likely contributed to a 22.9% increase in all-cause mortality in the US population from March 1, 2020, to January 1, 2021 [5].

The VHA is the largest integrated healthcare delivery system in the US serving 9 million enrollees in 2021 through 78.8 million ambulatory care visits [6]. The number of in person visits per month recorded in April 2020 was 532,827, down 89% from April 2019, reflecting the disruption to care due to COVID [7]. Pre-pandemic, VHA already had an established telehealth program and prioritized virtual care to promote access, especially to mental healthcare services [8]. The routine delivery of virtual primary and medical specialty care was also possible but was less prevalent across the national system.

Similar to other health care systems, the VHA system rapidly shifted to virtual care in the beginning of the pandemic [9]. In a retrospective study of VHA ambulatory care at one site early in the pandemic, there was a 56% decline in in-person visits, two-fold increase in telephone and video visits, and 30% overall reduction in outpatient visits [10]. In addition, a cross-sectional VHA-wide study found that in-person encounters declined more at VHA sites when compared to the community. This study also suggests that the VHA has been more conservative in reopening than community care providers, instead opting to spend more on community care [7].

While patients’ satisfaction with telemedicine has generally been high before and during the COVID-19 pandemic, there is little consensus regarding patients’ preferences for in person versus virtual visits [11, 12]. For example, one study of a large academic orthopedic practice did not find an association between patient satisfaction and mode of visit, while a large, representative survey of US adults found that participants were willing to engage with virtual care but preferred in person care [13, 14].

Little is known about the pandemic’s effects on the overall ambulatory care experience of veterans who primarily seek care within the Veterans’ Health Affairs (VHA) system, especially in the early part of the pandemic when there was so much uncertainty. We sought to characterize how the COVID-19 pandemic and the marked disruptions early in its course impacted the experience of veterans who utilize ambulatory care services in the VHA system especially in term of access, the ability to obtain healthcare services when needed [15], and satisfaction, the extent to which patients are content with the care received [16]. We also explored the context of veterans' daily lives during the pandemic, knowing that many people’s mental health, relationships, and employment were impacted. We suspected that these contextual factors impacted veterans’ lives and perceptions as much or more than systemic changes to the delivery of their health care.

## Methods

### Overview

After receiving institutional review board approval (IRB) from Baylor College of Medicine and VA Research and Development Committee approval, we performed a mixed-methods evaluation of access and satisfaction among current users of primary care. This was performed in partnership with primary care leadership of the Michael E. DeBakey VA Medical Center (MEDVAMC) in Houston, Texas. MEDVAMC is one of the largest and most complex facilities in the VHA providing quaternary services to more than 150,000 enrolled Veterans in Southeast Texas with robust education, training and research activities performed in conjunction with a closely affiliated academic medical center.

### Population and sampling

Eligibility criteria: Participants were selected from 31 primary care providers (PCPs) out of 40 at a single location. Provider panels were eligible if the provider was a full-time clinician with more than 80% clinical effort, defined by provider time, not panel size. Eligible patients had a scheduled appointment between March 1 and June 30, 2020, regardless of the outcome of that appointment (i.e., completed, cancelled, rescheduled, no-show) or the modality (i.e., in person, telephone, video). There were no exclusion criteria for the initial contact of patients; patients were excluded if they were unable to provide verbal consent in English at the time of the interview. Using an online random number generator, five patients were selected from each provider’s list with scheduled appointments during the period of interest and mailed them a letter explaining the project and how they could opt out. Trained team members called the patients approximately 10 days after the letters were mailed and requested verbal consent to conduct the semi structured interview. If a patient did not respond on first try, two additional attempts were made on subsequent business days at different times.

The questions were framed around the March-June 2020 period to coincide with the scheduled primary care appointments and at the beginning of the pandemic when Houston experienced its first wave of COVID infections. The number of infections in the Houston area increased from a total of 5 new cases on March 4th to almost 2,000 daily cases by June 30^th^ [17]. In mid-March, hospitals and other healthcare providers dramatically limited routine in-person clinical encounters and procedures, while still providing inpatient and emergency care; these restrictions were relaxed slightly in May and June. While the initial interviews were performed in July and August 2020, Houston infection rates decreased from 2,000 new cases on July 1st to 119 new cases on August 23^rd^. Ten additional interviews were conducted in September 2020, when new daily cases rose again, ranging from 58 to 443 [17]. No effective outpatient treatments were available for COVID infections during these periods other than supportive care, and no vaccines were yet available.

### Semi-structured interview guide

The semi-structured telephone interview guide consisted of a mix of 56 closed and open-ended questions in a patient-oriented sequence. The majority of the questions were adopted with appropriate modification from the VA Survey of Health Experiences of Patients (SHEP), which uses many items from the Hospital Consumer Assessment of Health Providers and Systems (HCAHPS), a healthcare industry standard [18]. SHEP is used to assess patients’ perception of their healthcare experience and includes topics such as ease of access to care and communication with providers. Testing of the interview guide indicated a completion time of less than 45 min including opportunities for patients to elaborate on their experiences and concerns. The interview guide included questions on: access (9 items), fragmentation (3 items), coordination (10 items), continuity (4 items), satisfaction/patient experience (13 items) and sociodemographic (2 items- health rating and insurance coverage) (See Additional file 1: appendix 1).

### Data collection

After verbal consent, co-authors (BT or AT) conducted the interview with the patient. The interviewer recorded responses and any additional notes during the interview into an excel database stored on a secured drive accessible only to specific research personnel. Interviewers read and recorded explicit item responses and wrote down verbatim quotes from the open-ended questions during the interview. “No response” was recorded when the question was not asked, e.g., if it wasn’t relevant to the participant’s situation or the context of the encounter made it irrelevant, or the participant refused to answer.

BT and AT also abstracted patient demographic data (e.g., age, sex, race/ethnicity, marital status, percentage of service-connected disability) from the VHA’s electronic medical record to supplement the interview data.

### Data analysis

Quantitative data were summarized using descriptive statistics, e.g., mean, standard deviation (SD), range, percentage. Not all items were asked of every participant, especially when the response to a previous question rendered an item not applicable, a participant declined to answer, or an interview ended prematurely; missing responses are denoted as “no response”. Responses to the open-ended questions and other narrative responses recorded during the interviews were extracted from the response database and compiled into a qualitative response database. Open-ended responses and veteran quotes were first assessed for over-arching themes by authors BT and AT after which authors BT, AT, and DAH employed a matrix analysis to further categorize the recorded quotes into over-arching themes [19]. Disagreement was resolved by consensus. Qualitative findings were further examined and triangulated with quantitative results with a focus on access, satisfaction, and comparisons of perceptions of VHA and non-VHA care changed during the pandemic.

## Results

Of 100 invitation letters sent to potential participants, zero opted out prior to phone contact. The team reached 49 potential participants of whom 40 consented to participate. Veterans were consented and included in our study on a rolling basis in July and August 2020 until we reached our initial saturation and recruitment goal of 30. After presenting preliminary findings to a community engagement committee composed of veterans, we altered our initial interview guide by adding questions specific to veterans’ perceptions of VHA vs. non-VHA care and removing questions found to be less relevant. We then completed an additional 10 interviews using the altered interview guide in September 2020. Three veterans (9%) terminated the interview prior to completion due to competing commitments.

### Demographics

The study sample was largely non-Hispanic men, almost equally split between White (52.5%) and African American (47.5%) racial categories. Approximately half (55%) of the study sample had a service-connected disability rating of 50% or greater, indicating the presence of a chronic condition caused or exacerbated during military service and the threshold for receiving comprehensive health at VHA at no cost. Nearly half (47.5%) of veterans rated their health as fair or poor. Many of the veterans had other insurance coverage, including TRICARE (*n* = 21 (52.5%)), Medicare (*n* = 8 (20%)), and private insurance (*n* = 5 (12.5%)) (Table 1).

**Table 1:** Participant characteristics (*n* = 40) from interviews and VHA medical record data

	**Mean (SD)**
Age (range 30 – 93 years)	62.3 (14.8)
	**N (%)**
Sex	
Male	27 (67.5)
Female	13 (32.5)
Race	
White	21 (52.5)
Black or African American	19 (47.5)
Ethnicity	
Not Hispanic or Latinx	33 (82.5)
Hispanic or Latinx	6 (15)
Unknown	1 (2.5)
Health Rating	
Excellent	1 (2.5)
Very Good	2 (5)
Good	18 (45)
Fair	13 (32.5)
Poor	6 (15)
Marital Status	
Married	6 (15)
Never married	14 (35)
Divorced	7 (17.5)
Separated	13 (32.5)
Widowed	3 (7.5)
Total Service Connection	
None	13 (32.5)
0 – 40%	5 (12.5)
50 – 90%	15 (37.5)
100%	7 (17.5)
Non-VHA Healthcare Insurance^a^	
Medicare	8 (20)
TRICARE	21 (52.5)
Private	5 (12.5)
None	6 (15)

*SD* Standard Deviation

^a^The non-VHA Healthcare Insurance is the only information obtained from the VHA medical record in this table

### Access

The number of self-reported completed primary care encounters per veteran was high during the reference period [mean 2.6 (SD 2.2)] and greater than the number reported as scheduled [mean 2.3 (SD 2.2)]; veterans indicated the use of same-day or urgent visits. Veterans utilized different modes for their total ambulatory (primary and specialty care) encounters: in-person [mean 1.9 (SD 1.6)], telephone [mean 2.1 (SD 1.8)], and video [mean 1.5 (SD 0.6)] (Table 2).

**Table 2:** Patient interview quantitative question responses (*N* = 40) reported by domains of access, satisfaction, and psychosocial effects of COVID

Survey Question	N (%)	Mean (SD)
**Access**		
Most recent encounter type?		
In-person	15 (37.5)	
Telephone	22 (55)	
Video	2 (5)	
No response	1 (2.5)	
Do you have a personal doctor you usually see if you need a checkup, want advice about a health problem, or get sick or hurt?		
Yes, VA	37 (92.5)	
Yes, non-VA	1 (2.5)	
Yes, both	1 (2.5)	
No	1 (2.5)	
Did you contact this doctor's office for an illness, injury, or condition that needed care right away?		
Yes	23 (57.5)	
No	16 (40)	
No response	1 (2.5)	
How many days have you had to wait for an appointment when you needed care right away?		
N/A (didn’t try to get an appointment)	11 (27.5)	
Same day	5 (12.5)	
1 day	4 (10)	
2–3 days	6 (15)	
More than 7 days	6 (15)	
No response	8 (20)	
How many PCP appointments did you have scheduled?		2.25 (2.2)
How many PCP encounters did you actually complete?		2.6 (2.2)
How many of each type of encounter did you complete?		
In-person		1.9 (1.6)
Telephone		2.1 (1.7)
Video		1.5 (0.6)
Compared to before the COVID-19 pandemic, have you had to miss more appointments than since the start of the pandemic and up to now?		
Yes	17 (42.5)	
No	19 (47.5)	
No response	4 (10)	
I had trouble scheduling my primary care appointments		
Yes (VA-related)	9 (22.5)	
Yes (non-VA related)	0	
No	27 (67.5)	
No response	4 (10)	
I waited too long to see the provider		
Yes (VA-related)	15 (37.5)	
Yes (non-VA related)	0	
No	22 (55)	
No response	3 (7.5)	
**Satisfaction**		
How does your satisfaction with the health care you have received compare with the time before start of COVID-19 pandemic?		
More satisfied	1 (2.5)	
The same	27 (67.5)	
Less satisfied	9 (22.5)	
No response	3 (7.5)	
**Psychosocial Effects**		
I felt anxiety over being exposed to or exposing others to COVID-19		
Yes	12 (30)	
No	26 (65)	
No response	2 (5)	
Have you received information about what to do if you need COVID-19 related care from your doctor?		
Yes (VA provider)	15 (37.5)	
Yes (non-VA provider)	0	
No	23 (57.5)	
No response	2 (5)	
Have you needed COVID-related care?		
Yes	1 (2.5)	
No	37 (92.5)	
No response	2 (5)	
Have you received COVID-related care?		
Yes	0	
No	24 (60)	
No response	16 (40)	

The majority of veterans (92.5%) reported having a VHA provider who serves as the primary care provider (PCP) from whom they usually seek care, while 2 veterans (5%) stated their usual PCPs were outside the VHA system. When asked about their most recent encounter with their PCP, 15 (37.5%) veterans spoke to their PCP in person, 22 (55%) by phone, and 2 (5%) by video. Thirteen veterans (33%) reported no change in their ability to see their PCP since the start of the pandemic.

A sizable minority (*n* = 9 (22.5%)) indicated more difficulty scheduling appointments, and 17 veterans (42.5%) reported a greater number of missed appointments between March and June 2020. Veterans were asked “How many days have you had to wait when you needed care right away”: 9 (22.5%) reported waiting “1 day” or “same day”, and 12 (30%) noted waiting 2 or more days. The remainder 19 (47.5%) did not seek care that was needed right away. When asked if their provider had “spent enough time with you” since the start of the pandemic, 9 veterans (22.5%) answered “no.”

Twenty-seven (67%) of the veterans reported decreased access to care through their comments. Matrix analysis of quotes from these 27 veterans showed 15 (56%) noted the theme of administrative barriers to access. These administrative barriers included frequent appointment changes, cancellations, and difficulty with phone communication (including long wait times, dropped, unanswered, and unreturned phone calls). One veteran illustrated these administrative barriers by saying, “I’d call the operator, I’d tell them my doctor’s name, they couldn’t find him, they would transfer me to the clinic, then be on hold at the clinic. Finally, the phone would hang up.” The next most cited barrier category (*n* = 9, 33%) was lack of provider availability with one veteran saying, “They are not reaching out like they used to. I used to love my doctors, but they are not on top of their job anymore. They give me less appointments, they forget about me.” Notably, 7 (26%) of the 27 veterans who reported challenges in access indicated that telehealth encounters were inadequate to meet their needs. One veteran said, “A lot of people aren’t getting the care they need. They need a more hands-on approach. You cannot take care of a person online”.

**Table 3:** Quotes from veterans grouped by theme. Italicized quotes are also reported in the text. Letters indicate specific veterans; multiple quotes were selected from some participants

**Access**	
**Decreased access**	
Personal barriers	“At the start, I did have to miss appointments because of my job being affected by the pandemic. I had to choose between missing work and missing appointments.”—Veteran A
Administrative barriers	*“I’d call the operator, I’d tell them my doctor’s name, they couldn’t find him, they would transfer me to the clinic, then be on hold at the clinic. Finally, the phone would hang up.”—Veteran B*
	It is a problem to have to drive over there and go through the hassle of waiting outside, being screened, and all of that. Sometimes when I think about having to go in, I just want to cancel.”—Veteran C
Lack of physician availability	*“They are not reaching out like they used to. I used to love my doctors, but they are not on top of their job anymore. They give me less appointments, they forget about me.”—Veteran D*
Waiting longer than normal to be seen	“They had to reschedule a colonoscopy 4–5 times. They had seen a few polyps last time so I really wanted to get it done but had to wait.”—Veteran E
Unable to get care	“I was supposed to get a mammogram but haven’t heard from anyone.”—Veteran F
	“I haven’t gotten needles in 3 months, I’m having to borrow my husband’s.”—Veteran D
**No change in access**	
	“It’s no different than it was before.”—Veteran G
	“The only difference is I have to talk to her on the phone instead of going in person.”—Veteran H
**Satisfaction**	
**Less satisfied**	
Understaffed	*“Since the pandemic, when I am on the phone with [my PCP] I can tell she seems overwhelmed and overworked. I just feel rushed whenever I am in the hospital.”—Veteran A*
	“From the time I called to when I spoke to a nurse, it was an hour and a half…They seem to be very behind and not very well staffed.”—Veteran I
	*“If I got sick I wouldn’t know what to do or where to go.”—Veteran J*
Telehealth inadequate to meet care needs	“I don’t do videos.”—Veteran K
	“The biggest difficulty has to be with my mental health. I am going to group therapy and it is over the phone now. It’s hard to connect and get all that I usually do out of the program. I would participate more if it were in person.”—Veteran A
	“I want a face to face with a doctor who will address my issues.”—Veteran I
	“Sometimes when someone calls to say it’ll be over the phone, I say “the hell with it.””—Veteran L
	*“A lot of people aren’t getting the care they need. They need a more hands-on approach. You cannot take care of a person online.”—Veteran M*
Expressed understanding about the changes	“It’s literally night and day, not for lack of caring on the part of the doctors and nurses.”—Veteran I
	*“This is all over the world. This isn’t just Houston. I understand why I can’t see [my PCP] right now.”—Veteran F*
	“I understand why things at the VA are worse but I’m still unhappy about it.”—Veteran B
**No change**	
	*“[My satisfaction] has not really changed…I know [my PCP] is busy, so I lay back until she calls me because I want those who are sick to get the treatment they need.”—Veteran N*
	“I am the type of person that I don’t really complain too much. I figure other people need help right now.”—Veteran O
	“I’m 100% disabled. I don’t have to be anywhere, do anything. I’ve just been staying at home. Things haven’t really changed.”—Veteran P
**VHA vs. non-VHA Care**	
**Access differences**	*“The accessibility is the reason I changed. Compared to the VA doctor, I believe he pays me more attention. I feel like the VA doctors have been stretched too thin for years. The primary care doctor on the outside seems to care for me as an individual…I have been very satisfied with all of the VA doctors, it’s just the accessibility that has been difficult…”—Veteran Q*
	“I have not had any problem with my primary care doctor or any doctor at the VA before the COVID pandemic. It is just hard to get in there…I feel like I have to see the non-VA doctor more because the VA people keep cancelling…I see doctors out of the VA more because it’s so hard to see VA doctors”—Veteran R
**Psychosocial Effects of the Pandemic**	
**Mental health**	*“I am extremely stressed out…Not sleeping right. Not eating right…This has been very hard…My mental health has taken a toll.”—Veteran I*
	“I have a history of PTSD and anxiety. It has my anxiety at a 10 everyday when I wake up, especially the uncertainty.”—Veteran A
	“Initially I was really depressed. I’ve been scared to be around other people…I have a lot of mental anguish.”—Veteran F
	“It’s a lot of stress being in the house cooped up…I would like to go to the park but I can’t walk with my condition. My PTSD is giving me crazy nightmares at night and that is added stress. I lost my…brother…and I couldn’t even go to his funeral.”—Veteran R
	“I have been depressed a lot lately, just want to be kinda to myself. I can cry at the drop of a dime. Once I get my daughter stabilized, I want to talk to my therapist.”—Veteran S
**Anxiety about spreading or catching COVID-19**	*“I suffer from anxiety attacks. It has increased since corona…I can’t be around anyone because I’m too sick.”—Veteran D*
**Social isolation**	“I feel like it has restricted me from seeing most of the people I am used to seeing…I stay inside and away from people.”—Veteran T
	*“The only thing that has been tough is the socialization. I feel like I am almost isolated at this point. I have a care group with vets for PTSD and I was attending that and it was helpful…We used to meet once a week. That has dried up and that is sorely missed.”—Veteran U*
	“I haven’t been able to counsel men at the Christian drug rehab. I have been home a lot.”—Veteran V
**Physical inactivity**	*“It has affected my activities. I used to go out to eat and I used to walk around the grocery store for exercise. I can’t do either of those now.”—Veteran Q*
**Financial changes**	“At the start, I did have to miss appointments because of my job being affected by the pandemic. I had to choose between missing work and missing appointments.”—Veteran A
	“I did get furloughed when it happened. My mom also stopped working. Besides our jobs, I think this has been a good thing. We have had time to focus on what is important and to become closer as a family.”—Veteran W

### Satisfaction

Most (31 (84%)) respondents were either “very satisfied” or “satisfied” with the health care received at their VA primary care facility during the period of interest. When asked to compare their satisfaction with their health care experience during the study period to before the pandemic 27 veterans (73%) felt it was “the same”, one (3%) was “more satisfied”, and nine (24%) were “less satisfied” with their experience. When veterans were asked to rate their VA primary care provider on a scale of 0–10, the majority (*n* = 21 (52.5%)) rated their provider 9 or 10 (mean 8.6 (SD 2.0).

Matrix analysis of direct quotes from the veterans in response to the question “How does your satisfaction with the health care you have received compare with the time before March 1st (start of COVID-19 pandemic)?” revealed that 25 (62.5%) did not have a change in their satisfaction with their health care since the pandemic, which is illustrated by this representative quote: “[My satisfaction] has not really changed…I know [my PCP] is busy, so I lay back until she calls me because I want those who are sick to get the treatment they need.” Two veterans (5%) reported they were dissatisfied with their care before the pandemic began, and eight (20%) veterans reported decreased satisfaction during the reference period. One veteran who reported decreased satisfaction said that “Since the pandemic, when I am on the phone with [my PCP] I can tell she seems overwhelmed and overworked. I just feel rushed whenever I am in the hospital.” However, seven (17.5%) veterans expressed understanding the reasons for the changes seen in accessing their health care due to the pandemic, with one veteran saying “This is all over the world. This isn’t just Houston. I understand why I can’t see [my PCP] right now.” Additional direct quotes presented in Table 3 illustrate these findings.

### VHA vs. non-VHA

Of 40 veterans surveyed, only one veteran utilized a non-VHA PCP and only one veteran had both VHA and non-VHA PCPs (only 2 (5%) saw a non-VHA PCP); 6 (15%) veterans saw non-VHA providers of any kind. For this reason, we focus on descriptive comparisons of VHA and non-VHA care only. When asked if their VHA PCP spent enough time with them, 68% of respondents responded “yes”, in comparison to both veterans (100%) who saw a non-VHA PCP. When asked if their VHA PCP “talked with you about specific goals for your health,” 64% of respondents said “yes,” whereas 83% of the six veterans who saw a non-VHA provider of any kind reported they talked to them about specific goals for their health (Table 4).

**Table 4:** Satisfaction and care experience of VHA users (*n* = 40) and non-VHA users (*n* = 6)

	**VHA users (*n* = 40)**	**Non-VHA users (*n* = 6)**
Using any number from 0 to 10, what number would you use to rate your < < VHA or non-VHA > > doctor?	8.6 mean (SD 2.1)	9.5 mean (SD 1.2)
Overall, how satisfied are you with the health care you have received at your < < VA or non-VA > > primary care facility since start of COVID-19 pandemic?		
*Very dissatisfied*	1 (2.5%)	-
*Dissatisfied*	2 (5%)	-
*Somewhat dissatisfied*	1 (2.5%)	1 (16.7%)
*Somewhat satisfied*	2 (5%)	-
*Satisfied*	9 (22.5%)	-
*Very satisfied*	22 (55%)	5 (83.3%)
*No response*	3 (7.5%)	-
Since March 1st (start of COVID-19 pandemic), has your < < VHA or non-VHA > > provider spent enough time with you?		
*Yes*	23 (57.5%)	6 (100%)
*No*	9 (22.5%)	-
*Not sure*	2 (5%)	-
*No response*	6 (15%)	-
Has anyone in your < < VHA or non-VHA > > provider team talked with you about specific goals for your health?		
*Yes*	21 (52.5%)	5 (83.3%)
*No*	12 (30%)	1 (16.7%)
* No response*	7 (17.5%)	-

*SD* Standard Deviation

Veterans were also asked to rate their VHA PCP on a scale of 1–10 (where 0 is the worst provider possible and 10 is the best provider possible). The mean rating of VHA PCPs was 8.6 out of 10 (SD 2.0, range = 0–10). We asked veterans to rate their non-VHA providers of any kind (due to the small number of veterans who saw a non-VHA PCP), and they rated them on average 9.5 out of 10 (SD 1.2, range = 7–10). When asked about their overall satisfaction with their VHA providers, 60% of respondents were “very satisfied” with their care, whereas 83% of veterans who had non-VHA providers of any kind were “very satisfied” with their non-VHA care.

When asked if they had received information about what to do if they needed COVID-related care, 15 (37.5%) of veterans reported that their VHA provider gave them this information, compared to none (0%) of veterans who saw non-VHA providers. Twenty-three (57.5%) veterans reported not receiving information about what to do if they needed COVID-related care from any doctor. One veteran said, “If I got sick I wouldn’t know what to do or where to go.”

From the responses to the open-ended questions, indications of relative dissatisfaction with VHA care compared to non-VHA care were mostly linked to perceived difference in attentiveness of VHA providers and the sense that non-VHA providers had greater bandwidth to provide individualized care. One veteran who utilized both VHA non-VHA providers said, “The accessibility is the reason I changed. Compared to the VA doctor, I believe he pays me more attention. I feel like the VA doctors have been stretched too thin for years. The primary care doctor on the outside seems to care for me as an individual…I have been very satisfied with all of the VA doctors, it’s just the accessibility that has been difficult…” Additional direct quotes located in Table 3 provide examples.

### Psychosocial effects of the COVID-19 pandemic

None of the veterans reported directly experiencing COVID-19 at the time of interview and only one (2.5%) veteran reported needing COVID-19 testing. Many of the veterans, however, spoke at length about changes to their everyday lives and mental health. Therefore, psychosocial factors related to the pandemic emerged as important context. In response to open-ended questioning, 6 (15%) veterans reported worsening of existing mental health conditions or the development of new mental health concerns. One veteran said, “I am extremely stressed out…Not sleeping right. Not eating right…This has been very hard…My mental health has taken a toll.” Twelve (30%) veterans felt anxiety about being exposed to or exposing others to the virus, with one saying, “I suffer from anxiety attacks. It has increased since corona…I can’t be around anyone because I’m too sick.” In addition, 8 (20%) veterans reported feeling more socially isolated and 3 (7.5%) reported being less physically active. One veteran with PTSD said, “The only thing that has been tough is the socialization. I feel like I am almost isolated at this point. I have a care group with vets for PTSD and I was attending that and it was helpful…We used to meet once a week. That has dried up and that is sorely missed.” Another veteran noted that “It has affected my activities. I used to go out to eat and I used to walk around the grocery store for exercise. I can’t do either of those now.” Additional direct quotes located in Table 3 illustrate these findings.

## Discussion

A combined analysis of the self-reported quantitative data and direct quotes from veterans provides a window into the effects of the early COVID-19 pandemic on the health care experience of veterans engaged in primary care at a large VA Medical Center. The veterans perceived new difficulty with accessing primary care despite frequent encounters, and a substantial minority expressed decreased satisfaction with their care at the beginning of the COVID-19 pandemic. In addition, veterans frequently reported psychosocial stressors related to the COVID-19 pandemic and their consequences, including worsening of mental health conditions and feelings of isolation.

We focused our analysis on access and satisfaction, as well as perceived differences between VHA and non-VHA care. Though the self-reported quantitative data did not suggest a decrease in access, but a shift to virtual means and urgent or same-day appointments, the narrative comments indicated veterans perceived more difficulty in accessing care during the first four months of the COVID-19 pandemic than prior to the pandemic. Explanations deduced from the interview themes included inadequate time with their provider, difficulty scheduling provider visits due to administrative barriers, and the inadequacy of telehealth services. The fact that a substantial minority (7 of 30 veterans) reported that telehealth did not meet their needs as discerned from qualitative analysis of their comments may have implications for the role of telemedicine more generally. The disjuncture between how healthcare organizations define and track access, e.g., through completed scheduled visits, may be substantially different from how patients talk about and experience access.

The VHA may wish to adjust its response to pandemics and other disasters to ensure a perception of continued access to care, in line with perceptions about access to private sector healthcare services [20]. However, this should be balanced by the fact that the COVID-19 pandemic, especially during the period referenced in this study, was associated with much uncertainty and healthcare facilities responded with the best information and approaches available to them at the time. Restricting access to ambulatory care was a widespread response to overwhelmed healthcare and important beneficial impact on other, more urgent demands, such as inpatient care of acutely and critically ill patients.

Due to stringent screening procedures and access protocols, only patients with scheduled appointments were allowed into the facility. No patient attendants were allowed with few exceptions (Personal communication with Himabindu Kadiyala, Director, PrimeCare April 19, 2021). This may have contributed to patients’ perceptions of lack of access. Satisfaction ratings implied that many of these barriers existed prior to, but were exacerbated by, the pandemic. The majority of veterans had no change in satisfaction in their overall care experience; however, a sizable minority were less satisfied. Of note, VHA providers were rated favorably overall.

While many of our veterans had access to non-VHA ambulatory care covered by TRICARE and Medicare, few reported utilizing these options, which is consistent with other reports [21]. Even with the COVID pandemic-related changes, only six veterans chose to receive care outside of the VHA system during the reference period. Direct quotes from veterans who sought non-VHA care suggested they did so because of perceived ease of access to non-VHA care and inadequate resources for care at the VHA. The frequency of non-VHA use in this sample may reflect a reliance on VHA care in this sample of sick, highly service-connected group of VHA users. For example, previous research showed that more than 60% of Medicare eligible older veterans with diabetes received at least some care from non-VHA providers [22]. The veterans who participated in this study included a relatively large portion of African Americans (48%) as compared to both the overall veteran population who utilize VHA care (about 15%) [23] and the general Houston population (20%) [24]. Given the disparities in access to private sector health care faced by people of color, this could have impacted their ability or choice to utilize VHA vs. non-VHA care [25].

On a scale from 0–10, six veterans rated their non-VHA providers 9.5 (median: 10, IQR: 9.25–10 compared to the 34 veterans who only used VHA PCPs and rated them an average of 8.6 (median: 9, IQR: 8–10). Also, a higher percentage of veterans who sought non-VHA care were ‘very satisfied’ with their experience. This could be due in part to the reported perception of more time spent with their non-VHA providers and their non-VHA providers more frequently addressing patient goals for their health. Our understanding of the relationship between VHA and non-VHA care was limited by the small number of veterans in our sample (6 of 40) who did seek care from non-VHA providers, but our findings suggest that veterans perceived non-VHA care to be more accessible and therefore more satisfactory during the early portion of the COVID pandemic. This is an important finding as there are few direct comparisons between VHA and non-VHA care during public health emergencies.

While none of our veterans had experienced COVID-19 at the time of the interviews, a substantial proportion of the veterans reported the exacerbation and/or development of mental health conditions such as anxiety and PTSD even without direct questioning. Veterans attributed this to multiple psychosocial stressors related to the pandemic, including less opportunities for social engagement (“I stay inside and away from people”) and physical activity, increased occupational stress and financial uncertainty (“I did get furloughed when [the pandemic] happened”), and general anxiety concerning the virus (“I suffer from anxiety attacks. It has increased since corona…”). Notably, in contrast to existing studies that espouse the benefits of telehealth [26, 27], a substantial proportion (17.5%) of the veterans did not feel that telehealth was meeting their healthcare needs, with some specifically mentioning their mental health care. In addition, the majority of veterans reported not receiving guidance from a provider on how to seek COVID-19-related care, perhaps contributing to their feelings of uncertainty. Of note, however, more veterans reported receiving this information from VHA providers than non-VHA providers.

During this time, the local VA medical center was communicating daily COVID-related updates, guidance to access care, and resources through its website, social media accounts, and occasional text messages to registered VHA users (Personal communication with Maureen Dyman, Public Affairs Officer, August 20, 2020). Given the high prevalence of mental health conditions among veterans who use VHA primary care, exploring ways to enhance communication about accessing care, including virtual mental health, represents an important opportunity to improve the veteran care experience.

VHA PCPs, nurses and support staff were pulled from primary care responsibilities to augment several other critical COVID-related care responses, including staffing the inpatient COVID service and public health screening activities at the campus (Personal communication with Himabindu Kadiyala, Director, PrimeCare, April 19, 2021). These activities created real shortages among primary care personnel which were felt by our respondents. Some veterans recognized the cause of the decreased access to primary care, but not all. Once again, VHA communicated extensively about availability of services and how to receive urgent and emergent care and encouraged telephone and video modalities for more routine encounters to overcome the loss of primary care capacity and restricted physical access. It seems that even though there was a lot of general, institutional messaging from VHA, this did not translate to Veterans feeling like they knew how to more effectively navigate new COVID protocols, and these communications could not replace what the more frequent, personalized, and direct messaging from providers they received pre-COVID.

Our findings suggest several opportunities to improve the veteran care experience. While most veterans were able to access care from the VHA during the pandemic, as evidenced by the report of completed encounters, there was a general sense of difficulty in navigating new protocols and channels for receiving care, and some dissatisfaction with receiving care remotely. Perhaps, a major priority of the VHA system in situations of uncertainty related to public health emergencies is to expand communication efforts and enhance veterans’ perception of access.

This study suggests that the VHA system can benefit veterans through more streamlined, timely, and consistent communication with veterans. In addition to the online and social media presence, more robust telephone triage and response might address concerns we heard about dropped, unanswered, and unreturned phone calls, for example. Given the age, multiple chronic illnesses, and mental health issues of the population, the telephone call center response may be the most important means of reassuring and assisting veterans.

Our study highlights the importance of the psychosocial impact of COVID-related factors that impact veterans’ lives and may color their healthcare experience. Some of these factors are outside the scope of the VHA system but given the high prevalence of mental health conditions among veterans who use the VHA, the VHA system could help veterans by further expanding the visibility and reach of their virtual mental health care services, which were in fact bolstered during the pandemic. Lessons from this pandemic could be used to better advertise, communicate, and engage veterans on various virtual platforms to more fully meet their care needs.

Finally, while efforts were made by the VHA system to communicate with veterans about the pandemic – apparent from the results given the higher percentage of VHA providers communicating about COVID-19 compared to non-VHA providers – many veterans still perceived a lack of communication. Future work could explore how the content, framing, and timing of these communications impact perceptions of access and satisfaction.

Strengths of our study include our study sample composed of ‘real world’ veterans, which approximates the local VHA user population. Our findings are enhanced by the combination of quantitative and qualitative results. Further, incorporating the feedback provided by the veteran community engagement committee into the research activities enhanced the rigor and relevance of the work. Limitations include this being a cross-sectional study ‘look back’ interview with self-reported information, which makes our results susceptible to recall bias. We were unable to use medical record data to verify Veteran-reported healthcare utilization resulting in the possibility of inaccurate or biased reporting. This is less likely given the short time from the period of interest (March-June 2020) and the interviews (July–September 2020). Our results may not be generalizable to the larger veteran population due to the small number of veterans, who all sought care at a single site. The large and complex nature and urban context of the study site also limit generalizability of these findings.

## Conclusion

The findings of this study serve to illustrate the importance of perceived access and communication among the veteran population, especially during times of increased uncertainty and social stress. Though the quantitative data suggests continued adequate access and satisfaction, the frequent comments regarding barriers to care illustrate a disconnect between veterans’ perceived experience and the quantitative findings. Findings pertaining to virtual appointments, health information messaging, and mental health engagement during the early phases of the COVID-19 pandemic can be used to improve the overall care experience for VHA users and other patients, particularly during public health emergencies.

## Supplementary Information

**Additional file 1: Appendix 1.**

## Data Availability

Please contact the corresponding author for information on how to access the datasets analyzed during the current study.

## Abbreviations

COVID-19: Coronavirus Disease 2019
VHA: Veterans’ Health Administration
IRB: Institutional Review Board
VA: Veterans Affairs
PCP: Primary Care Provider
BT: Brice Thomas
AT: Aanchal Thadani
DAH: Drew A. Helmer
PVC: Patricia V. Chen
LMK: Lisa M. Kern
SHEP: Survey of Health Experiences of Patients
HCAHPS: Hospital Consumer Assessment of Health Providers and Systems
CPRS: Computerized Patient Record System
PTSD: Post-traumatic Stress Disorder
SD: Standard Deviation
