# Supplementary material for: Veterans’ ambulatory care experience during COVID-19: veterans’ access to and satisfaction with primary care early in the pandemic
Source: BMC Prim Care. 2022 Sep 21;23:245. doi: 10.1186/s12875-022-01851-3 (PMC9491256; doi:10.1186/s12875-022-01851-3)
Supplement: Supplementary file 1 — Additional file 1: Appendix 1. [file 12875_2022_1851_MOESM1_ESM.docx]

**Appendix 1**

| Question | Domain | Source | Survey Version |
| --- | --- | --- | --- |
| 1.     In general, how would you rate your overall health? |  | SHEP | 1 & 2 |
| 2.     Which type of health insurance do you have? |  | SHEP | 1 & 2 |
| 3.     When was the last time you were able to speak with any doctor about your care? | Access | SHEP | 1 & 2 |
| a.     Was it a VA or non-VA doctor? | Access | New | 1 & 2 |
| b.     Was this interaction in person, on the telephone, or via video call? | Access | New | 1 & 2 |
| c.     Was this with a primary care practitioner, specialist, or emergency practitioner? | Access | New | 1 & 2 |
| 4.     Do you have a personal doctor you usually see if you need a check-up, want advice about a health problem, or get sick or hurt? | Access | SHEP | 1 & 2 |
| 5.     When was the last time you sought care from a doctor outside the VA health system? | Fragmentation | New | 2 only |
| 6.     Have you ever sought care in the community paid for by the VA (CHOICE Act or MISSION Act)? Why or why not? | Fragmentation | New | 2 only |
| 7.     When was the last time you received care in the community paid for by the VA? | Fragmentation | New | 2 only |
| 8.     Has your ability to receive care in the community paid for by the VA changed during COVID? In what ways? | Access | New | 2 only |
| 9.     Did you contact this provider's (your primary care doctor) office to get an appointment for an illness, injury, or condition that needed care right away? | Access | SHEP | 1 & 2 |
| 10.  How many days have you had to wait for an appointment when you needed care right away? | Access | SHEP | 1 & 2 |
| 11.  How many appointments did you have scheduled? | Continuity | SHEP | 1 & 2 |
| 12.  How many of those visits were with your usual doctor? | Continuity | SHEP | 1 & 2 |
| 13.  How many of those encounters did you actually complete? | Continuity | SHEP | 1 & 2 |
| In-person? Telephone? Video? | Continuity | New | 1 & 2 |
| 14.  In your own words, how has your ability to see your usual primary care doctor changed from normal since the start of the COVID-19 pandemic? | Access, Continuity | New | 1 & 2 |
| 15.  Have you used secure messaging online to contact this provider's office? | Access | New | 1 & 2 |
| 16.  When you contacted this provider's office using secure messaging, how often did you get a helpful response as soon as you needed? | Access | New | 1 & 2 |
|  |  |  |  |
| The next set of questions will be asking about visits with doctors OTHER THAN your primary care doctor. |  |  |  |
|  |  |  |  |
| 17.  How many doctors’ visits did you have scheduled with a provider who is NOT your primary care doctor? | Fragmentation | SHEP | 1 & 2 |
| Specialty? Setting? | Fragmentation | SHEP | 1 & 2 |
| 18.  How many of those encounters did you actually complete? | Fragmentation | SHEP | 1 & 2 |
| In-person? Telephone? Video? | Fragmentation | New | 1 & 2 |
|  |  |  |  |
| The next set of questions will be asking about your experiences with your health care overall. |  |  |  |
|  |  |  |  |
| 19.  Compared to before the COVID-19 pandemic, do you feel you have seen more healthcare providers than normal? | Fragmentation | New | 1 & 2 |
| 20.  Compared to before the COVID-19 pandemic, have you had to miss more appointments than since the start of the pandemic and up to now? | Fragmentation | New | 1 & 2 |
| 21.  16. Have you experienced any difficulties regarding your health care since the start of the COVID-19 pandemic? Can you tell me about them? | Access | New | 1 & 2 |
|  |  |  |  |
| The next set of questions will be asking if you had trouble with some specific situations regarding your health care. |  |  |  |
|  |  |  |  |
| 22.  Have you experienced any of the following scenarios? | Access | SHEP | 1 & 2 |
| 23.  I had trouble scheduling my primary care appointments. | Access | SHEP | 1 & 2 |
| 24.  I had trouble scheduling my appointments with specialists. | Access | SHEP | 1 & 2 |
| 25.  I had difficulty traveling to my appointments. | Access | SHEP | 1 only |
| 26.  I felt anxiety over being exposed to or exposing other to COVID-19. | Access | SHEP | 1 & 2 |
| 27.  I waited too long to see the provider. | Access | SHEP | 1 & 2 |
| 28.  I had difficulty obtaining my prescription. | Access | SHEP | 1 only |
| 29.  I had difficulty picking up my prescription. | Access | SHEP | 1 only |
| 30.  I had difficulty paying for my prescription. | Access | SHEP | 1 only |
| 31.  How have those experiences been compared to before the COVID-19 pandemic? | Access | SHEP | 1 & 2 |
| 32.  Since March 1st, did your provider order a blood test, x-ray, or other test for you? | Coordination | SHEP | 1 & 2 |
| 33.  When this provider ordered a blood test, x-ray, or other test for you, did someone from this provider's office follow up to give you those results? | Coordination | SHEP | 1 & 2 |
| 34.  Have you or someone from this provider's office talked about all the prescription medicines you were taking? | Coordination | SHEP | 1 & 2 |
| 35.  Did you share any VA health record information you accessed through My HealthEVet with a community (non-VA) health care provider? | Coordination | SHEP | 1 & 2 |
| 36.  How did sharing this information impact your care with your community (non-VA) provider(s)? | Coordination | SHEP | 1 & 2 |
| 37.  Have you received information about what to do if you need COVID-19 related care from your doctor? (What information would have been helpful?) | Coordination | New | 1 & 2 |
| 38.  Have you received any text communication related to COVID-19 from your provider? | Coordination | New | 1 & 2 |
| 39.  Did you find the text communication helpful? | Coordination | New | 1 & 2 |
| 40.  Have you needed COVID-19-related care? | Access | New | 1 & 2 |
| 41.  Have you received COVID-19-related care? | Access | New | 1 & 2 |
|  |  |  |  |
| The next set of questions will again be asking about your experiences with your primary care doctor. |  |  |  |
|  |  |  |  |
| 42. Since March 1st, has Dr. (VA provider associated with patient) spent enough time with you? | Satisfaction | SHEP | 1 & 2 |
| 43. [for VA provider] Has anyone in this provider's office talked with you about specific goals for your health? | Satisfaction | SHEP | 1 & 2 |
| 44. [for VA provider] Using any number from 0-10, where 0 is the worst provider possible, what number would you use to rate this provider? | Satisfaction | SHEP | 1 & 2 |
| 45. [for VA provider] Have the clerks and receptionists at this doctor's office been as helpful as you thought they should be? | Satisfaction | SHEP | 1 only |
| 46. [for VA provider] Have the clerks and receptionists at this provider's office treated you with courtesy and respect? | Satisfaction | SHEP | 1 only |
| 47. IF PATIENT HAS SEEN A NON-VA PROVIDER: Did your non-VA provider spend enough time with you? | Satisfaction | SHEP | 1 & 2 |
| 48. Has anyone in this provider's office talk with you about specific goals for your health? | Satisfaction | SHEP | 1 & 2 |
| 49. Using any number from 0-10, where 0 is the worst provider possible, what number would you use to rate this provider? | Satisfaction | SHEP | 1 & 2 |
| 50. Have the clerks and receptionists at this provider's office been as helpful as you thought they should be? | Satisfaction | SHEP | 1 only |
| 51. Have the clerks and receptionists at this provider's office treated you with courtesy and respect? | Satisfaction | SHEP | 1 only |
| 52. Overall, how satisfied are you with the health care you have received at your VA primary care facility since March 1st? | Satisfaction | SHEP | 1 & 2 |
| 53. Overall, how satisfied are you with the health care you have received at your non-VA provider? | Satisfaction | SHEP | 1 & 2 |
| 54. How does you satisfaction with the health care you have received compare with the time before March 1st? | Satisfaction | New | 1 & 2 |
| 55. How is the COVID-19 pandemic affecting you and those close to you? |  | New | 1 & 2 |
| 56. Is there anything you feel you need to better take care of yourself during this time? |  | New | 1 & 2 |
